# Synergistic Regulation of Microglia Gene Expression by Natural Molecules in Herbal Medicine

**DOI:** 10.1155/2021/9920364

**Published:** 2021-08-18

**Authors:** Qiburi Qiburi, Temuqile Temuqile, Huricha Baigude

**Affiliations:** ^1^Institute of Mongolian Medicinal Chemistry, School of Chemistry and Chemical Engineering, Inner Mongolia University, Hohhot 010020, Inner Mongolia, China; ^2^International Hospital of Mongolian Medicine, Hohhot 010021, Inner Mongolia, China

## Abstract

The activated microglia contribute to stroke-induced neuroinflammation by upregulating the expression of a pleura of genes that are characterized as either proinflammatory or anti-inflammatory. The natural products alantolactone (Ala) and dehydrodiisoeugenol (Deh) found in *Inula helenium* L. and *Myristica fragrans* Houtt., respectively, are regularly used in traditional herb medicine, which play anti-inflammatory and antioxidant roles via regulation of canonical pathways such as nuclear factor kappa B (NF-*κ*B) in microglia and microphages. To illustrate the full spectra of gene expression alteration in microglia treated with Ala, Deh, and the mixture of Ala and Deh (denoted as Mix), we performed RNA-seq analysis of total RNA extracted from lipopolysaccharide- (LPS-) treated microglia subsequently exposed to Ala, Deh, and Mix. While both chemicals regulated the gene expression that facilitates an anti-inflammatory polarization, the mixture exerted some distinctive synergic regulatory effect, which differed from either of the chemicals alone. Our data provide important evidence for further research on the therapeutic mechanism of traditional medicine including Eerdun Wurile (EW).

## 1. Introduction

Microglia are the main immune cells in the central nervous system (CNS) [1, 2]. In a normal brain, resting microglia exhibit a ramified phenotype and play an important role in monitoring the brain parenchyma and interacting dynamically with neighboring neurons, astrocytes, and blood vessels [3, 4]. After the brain injury, microglia are immediately activated and polarized toward a classically activated (M1, proinflammatory) phenotype and alternatively activated (M2, anti-inflammatory) phenotype [2, 5, 6]. They can also convert their phenotypes dynamically [6]. M1 phenotype microglia can produce a variety of mediators, including inducible nitric oxide synthase (Nos2), C-X-C motif chemokine 10 (Cxcl10, as known as IP10), tumor necrosis factor (Tnf*α*), and cyclooxygenase-2 (Cox2) to induce oxidative stress, inflammation, and necroptosis after stroke [6]. For example, TNF*α*, a proinflammatory cytokine, causes brain damage through nuclear factor kappa B (NF-*κ*B) and mitogen-activated protein kinase (MAPK) signaling pathways to release proinflammatory cytokines and increase excitotoxicity [6]. Similarly, Il1*β* [6] and Cxcl10 [7] are also regulated by the NF-*κ*B signaling pathway to cause neuronal apoptosis. Various reports suggest that the modulation of microglia-derived cytokines and neuroinflammatory proteins may play a neuroprotective role in brain injury [8–11].

In addition, it has been found that free radical damage (reactive oxygen/nitrogen species, ROS/RNS) also plays a key role in diseases such as stroke, brain traumatic injury, and Alzheimer's disease (AD). ROS/RNS can lead to DNA damage, lipid peroxidation, and protein dysfunction, resulting in cell damage and death [12, 13]. When ischemic injury occurs in the brain, it will lead to neuroinflammation, and immune cells produce a large amount of ROS, which aggravates oxidative stress injury. ROS can also activate these inflammatory cells. For example, ROS activates microglia, neutrophils, and macrophages through the NF-*κ*B pathway [12]. Therefore, antioxidants are being studied as neuroprotective therapeutic agents for brain injury.

The therapeutic effect of herbal medicine such as traditional Chinese medicine (TCM) originates from the synergic power of multiple ingredients. For example, the active chemical ligustrazine in Bizheng-Tang, an eight-component TCM, in combination with leflunomide can alter the gene expression pattern in rheumatoid arthritis (RA) and overcome the low response of RA treatment to leflunomide alone. Previously, we fractionated and analyzed the active chemicals in the traditional Mongolian medicine Eerdun Wurile (EW) and discovered several immunomodulatory small molecules such as alantolactone, dehydrodiisoeugenol, myristicin, and costunolide that suppress proinflammatory polarization of microglia and might contribute to the overall neuroprotective activity of EW [14, 15]. Alantolactone (Ala) is one of the key bioactive molecules discovered in *Inula helenium* L., which is used as an important ingredient of EW. Ala exhibits antineuroinflammatory effects through suppression of NF-*κ*B and MAPK pathways [16, 17]. Similarly, dehydrodiisoeugenol (Deh), the main active component of *Myristica fragrans* Houtt., another key material of EW, has been reported to inhibit the expression of NF-*κ*B and other genes involved in inflammation in macrophages [18].

In the middle cerebral artery occlusion and reperfusion (MCAO/R) rat model, Ala could inhibit the expression of proinflammatory factors via suppression of NF-*κ*B and MAPK signaling pathways [17] and had neuroprotective effect in traumatic brain injured rats [16]. In cigarette smoke-induced human bronchial epithelial cells, Ala inhibited inflammation, apoptosis, and oxidative stress by activating Nrf2/HO-1 and inhibiting the NF-*κ*B pathway [19]. In RAW264.7 cells, Deh inhibits LPS-stimulated NF-*κ*B activation and Cox2 expression [18]. Deh showed high hydroxyl radical scavenging activity and DPPH radical scavenging activity [20].

We hypothesize that the neuroprotective effect of EW results from the synergic effect of active chemicals including Ala and Deh. To assess the combinatory effect of Ala and Deh on microglia polarization, in this report, we investigated the impact of each single molecule as well as the mixture on the gene expression pattern of BV2 cells, focusing on the inflammation pathways. We used RNA-seq technology to confirm the synergistic regulation of gene expression in LPS-stimulated microglia by natural products, Ala and Deh. Significant differential expression was discovered in the synergistic effect of the two bioactive molecules, including anti-inflammatory and antioxidant effects, which may provide the neuroprotective mechanism of EW.

## 2. Materials and Methods

### 2.1. Chemicals and Reagents

Alantolactone (catalogue no. A114070) was purchased from Aladdin, China. Dehydrodiisoeugenol (catalogue no. B21021) was purchased from Shanghai Yuanye Bio-Technology Co., Ltd (Shanghai, China). Lipopolysaccharide (LPS) (catalogue no. L2880) was purchased from Sigma-Aldrich, China. DMEM (high glucose) was purchased from Biological Industries, Kibbutz Beit Haemek, Israel. FBS (HyClone) and penicillin/streptomycin were purchased from Gibco BRL, Grand Island, NY, USA. 3-(4,5-dimethylthiazol-2-yl)-5-(3-carboxymethoxyphenyl)-2-(4-sulfophenyl)-2H-tetrazolium, inner salt (MTS) assay was purchased from Promega, Madison, WI, USA. TRIzol Reagent was purchased from Invitrogen, Carlsbad, CA, USA. PrimeScript™ RT Master Mix (Perfect Real Time) and TB Green™ Premix Ex Taq™ II (Tli RNase H Plus) and primers were purchased from Takara Biotechnology (Dalian) Co., Ltd, Dalian, China.

### 2.2. Cell Culture

In this study, the mouse BV2 microglial cell lines were bought from Shanghai Zhong Qiao Xin Zhou Biotechnology Co., Ltd, Shanghai, China. Cell lines were maintained in DMEM (high glucose) supplemented with 10% FBS (HyClone), 1% 100 units/mL penicillin, and 100 *μ*g/mL streptomycin at 37°C in humidified 5% CO_2_.

### 2.3. Measurement of Cell Viability

Cell viability was evaluated by 3-(4,5-dimethylthiazol-2-yl)-5-(3-carboxymethoxyphenyl)-2-(4-sulfophenyl)-2H-tetrazolium, inner salt (MTS) assay according to the manufacturer's specifications. BV2 cells were cultured in 96-well plates at 1 × 10^4^ cells/well in 100 *μ*L of culture medium until 70%–80% confluency was reached for experimental use. All of the alantolactone (Ala) and dehydrodiisoeugenol (Deh) were dissolved in DMSO, and all stock solutions were of 100.0 mM and 1.0 mM, respectively. Cells were treated with different concentrations of Ala (4.3 *μ*M, 21.5 *μ*M, 43.0 *μ*M, 64.5 *μ*M, and 107.5 *μ*M) and Deh (0.1 *μ*M, 0.2 *μ*M, 0.5 *μ*M, 1.0 *μ*M, and 2.0 *μ*M). After 24 h of incubation, 20 *μ*L of MTS was directly added to each well of the assay plate containing the samples in 100 *μ*L of culture medium. The plate was incubated for 1 to 4 hours at 37°C. The optical density (OD) of the samples was measured on a plate reader at 450 nm, and the results were expressed as a percentage of nontreated group.

### 2.4. RNA Isolation and Library Construction for Illumina Sequencing

BV2 cells were cultured in a 24-well plate at 5 × 10^4^ cells/well and were pretreated with 21.5 *μ*M Ala, 1.0 *μ*M Deh, and the mixture of 21.5 *μ*M Ala and 1.0 *μ*M Deh (denoted as “Mix”) for 12 h. Then, the cells were stimulated with LPS (100 ng/mL) in the presence or absence of Ala (or Deh or Mix). After 6 h of LPS treatment, total RNA was extracted from BV2 cells using TRIzol Reagent according to the manufacturer's specifications. The high purity (28S/18S ≥ 2.0) and high integrity (RIN >8.0) of RNA were evaluated using the Agilent 2100 bioanalyzer. The extracted total RNA was stored at −80°C and subsequently used for RNA-seq and RT-qPCR analyses.

The total RNA was extracted, and mRNA of BV2 cells was purified by using the oligo(dT)-attached magnetic beads. Adding the fragmentation reagent, the mRNA was interrupted to small pieces, then the first strand cDNA was synthesized by random hexamer-primer reverse transcription, and then the second-strand cDNA was synthesized. The double-stranded cDNA was subjected to end repair and adenylated the 3′ ends. Finally, adaptors were ligated to the cDNA fragments. The required fragments were purified by AMPure XP beads (AGENCOURT) and enriched by PCR amplification. The sample library was validated on the Agilent Technologies 2100 bioanalyzer.

### 2.5. Sequence Filtering, Mapping, and Assembly

Raw sequences were filtered with SOAPnuke (v1.5.2) [21] through removing aggressive adaptors, ploy-N and low-quality reads, and clean reads were stored in the FASTQ format. The clean reads were mapped to the mouse genome reference (Mus_musculus, reference genome version: GCF_000001635.26_GRCm38.p6) using HISAT2 (v2.0.4) software [22] and aligned to the reference coding gene set using Bowtie2 (v2.2.5) [23]. RSEM (v1.2.12) [24] was used to calculate the expression level of genes.

### 2.6. Differential Expression Analysis

Analysis of two groups' differential expression gene was performed using DESeq2. The expression level of the transcript was calculated in fragments per kilobase million (FPKM). Genes with FDR ≤0.05 found by DESeq2 were assigned as differentially expressed. For an optimal comparison of the results, cluster analysis was performed on the differential genes of NT vs LPS, LPS vs Ala, LPS vs Deh, and LPS vs Mix.

### 2.7. GO and KEGG Enrichment Analysis of Differentially Expressed Genes

In order to systematically analyze the DEGs function of our data, we used Phyper (https://en.wikipedia.org/wiki/Hypergeometric_distribution) to perform Gene Ontology (GO) (http://www.geneontology.org/) and Kyoto Encyclopedia of Genes and Genomes (KEGG) (https://www.kegg.jp/) enrichment analysis of annotated DEGs based on the hypergeometric test. The FDR ≤ 0.05 was regarded as the significant level of terms and pathways.

### 2.8. Quantitative Real-Time PCR (RT-qPCR) Analysis

Quantitative real-time PCR (RT-qPCR) was used to verify the reliability of high-throughput sequencing. The same RNA samples were used for RT-qPCR analysis and RNA-seq experiment. 0.5 µg total RNA was reverse-transcribed using PrimeScript™ RT Master Mix (Perfect Real Time), and quantitative PCR was performed on TB Green™ Premix Ex Taq™ II (Tli RNase H Plus). The sequences of primers used for RT-qPCR were as follows: mouse *β-actin*, forward: 5′-ctaaggccaaccgtgaaaag-3′, reverse: 5′-accagaggcatacagggaca-3′; mouse *Csf*3, forward: 5′-gctgctggagcagttgtg-3′, reverse: 5′-ttgacatagcagcatgtggat-3′; mouse *Ccl*7, forward: 5′-ttctgtgcctgctgctcata-3′, reverse: 5′-ggtctgggccatagaactga-3′; mouse *Ccl*2, forward: 5′-catccacgtgttggctca-3′, reverse: 5′-gatcatcttgctggtgaatgagt-3′; mouse *Il*1*rn,* forward: 5′-ctccttctcatccttctgtttca-3′, reverse: 5′-ggtcttctggttagtatcccagatt-3′; mouse *Il*1*β*, forward: 5′-agttgacggaccccaaaag-3′, reverse: 5′-agctggatgctctcatcagg-3′; mouse *Nos*2, forward: 5′-ctttgccacggacgagac-3′, reverse: 5′-tcattgtactctgagggctgac-3′; mouse *Tnfα*, forward: 5′-tcttctcattcctgcttgtgg-3′, reverse: 5′-ggtctgggccatagaactga-3′; mouse *Cxcl*10, forward: 5′-gctgccgtcattttctgc-3′, reverse: 5′-tctcactggcccgtcatc-3′; mouse *Blvrb,* forward: 5′-ggggccatcctgaaactc-3′, reverse: 5′-ggctggggtccttctgac-3′; mouse *Prdx*1, forward: 5′-gcactggaccatttttctgc-3′, reverse: 5′-ggcttatctggaatcacacca-3′; mouse *Cat,* forward: 5′-gtgcatgcatgacaaccag-3′, reverse: 5′-tgaagcgtttcacatctacagc-3′; mouse *Srxn*1, forward: 5′-aggggcttctgcaaaccta-3′, reverse: 5′-tggcatagctacctcactgct-3′; mouse *Slc*48*a*1, forward: 5′-cggtccttagggaattgaca-3′, reverse: 5′-tggattctgtccaactgtgc-3′; mouse *Sqstm*1, forward: 5′-ttcaaaagaagtggacccatc-3′, reverse: 5′-tgggagagggactcaatcag-3′.

### 2.9. Statistical Analysis

The data were analyzed using GraphPad Prism 7.0. All the results were expressed as mean ± SD. Statistical significance was determined using one-way ANOVA with Dunnett's multiple comparisons test. *P* < 0.05 was considered statistical significance.

## 3. Results

### 3.1. Cell Viability

**Figure 1:**
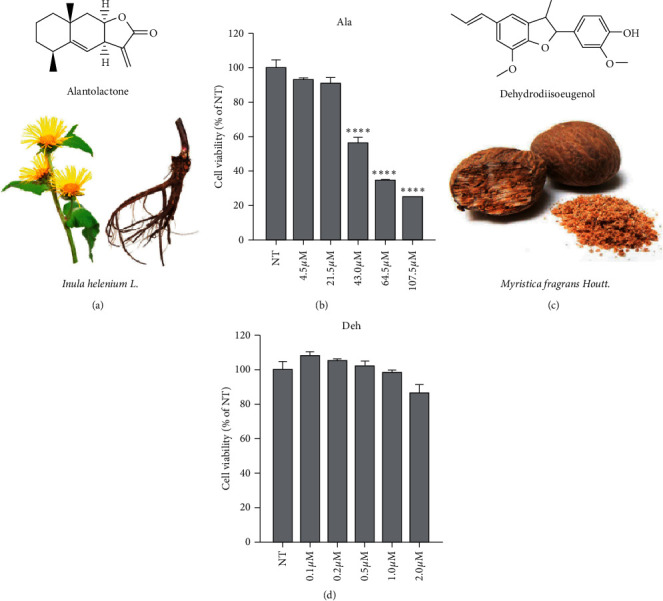
The plant origin and chemical structure of alantolactone (Ala) and dehydrodiisoeugenol (Deh) and the cytotoxicity assessment of Ala and Deh. Cell viability was performed on 1 × 10^4^ BV2 (mouse microglial cell lines) cells/well in 100 *μ*L of DMEM medium supplemented with 10% FBS and 1% penicillin-streptomycin. All of the alantolactone (Ala) and dehydrodiisoeugenol (Deh) were dissolved in DMSO, and all stock solutions were of 100.0 mM and 1.0 mM, respectively. Cells were treated with different concentrations of Ala and Deh. MTS assay showing the cell viability of BV2 cells treated with different concentrations of Ala and Deh. Results were expressed as mean ± S.D (*n* = 3). (a, b) Ala; (c, d) Deh. ^∗∗∗∗^P <0.0001 indicates statistical significance.

The plant origin and structure of alantolactone (Ala) and dehydrodiisoeugenol (Deh) are shown in Figure 1. To obtain the optimal concentration of the compounds for further in vitro assay, the MTS assay was performed in this experiment. The results showed that Ala and Deh did not impact the BV2 cells (mouse microglia cell line) viability at concentrations 21.5 *μ*M and 1.0 *μ*M, respectively (*P* < 0.05) (Figure 1). Therefore, the concentrations of 21.5 *μ*M Ala and 1.0 *μ*M Deh were used for the following experiment.

### 3.2. Illumina Sequencing and Aligning to the Reference Genome

In this study, RNA-seq was used to analyze variations in gene expression of the BV2 cells transcriptome. We sequenced 10 cDNA libraries (NT, LPS, Ala, Deh, and Mix) using Illumina (paired-end) sequencing technology and obtained 47.15 million raw reads on average. After removal of low-quality reads (quality scores <15), adaptor sequences, and ambiguous reads, 90.89% of the clean reads were aligned to the reference genome using Bowtie2. 44,887,103 (NT), 45,119,346 (LPS), 44,717,416 (Ala), 44,972,023 (Deh), and 44,763,779 (Mix) clean reads were retained for further mapping and differential expression analysis. The summary of sequencing quality and mapping is shown in Table 1.

### 3.3. Differential Expressed Genes (DEGs) after Ala, Deh, and Mix Treatment

**Figure 2:**
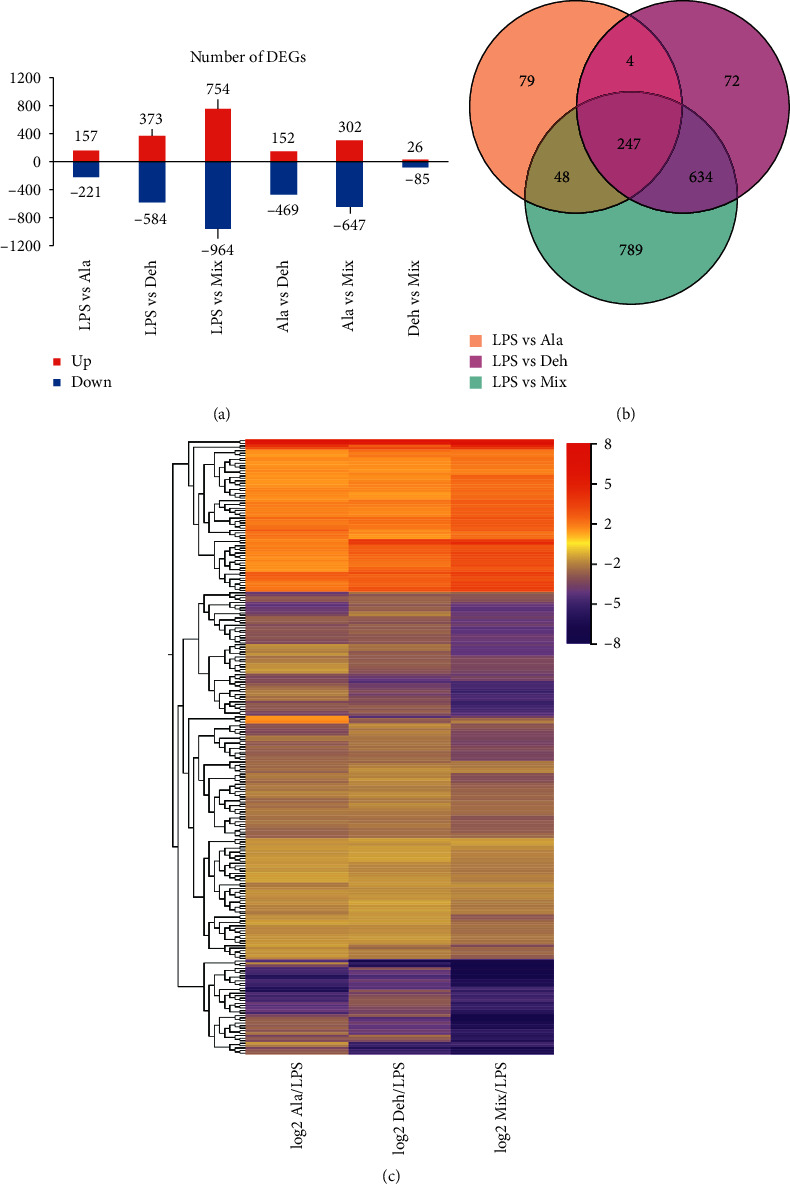
Analysis of gene expression based on |log2FC| ≥1 and FDR ≤0.05. (a) The bar graph showing the number of up- and downregulated DEGs. In LPS vs Ala (Ala group), 157 upregulated and 221 downregulated DEGs; 373 upregulated and 584 downregulated DEGs were found in LPS vs Deh (Deh group); and 754 upregulated and 964 downregulated DEGs were found in LPS vs Mix (Mix group). (b) Venn diagram showing the overlap of DEGs. Among these DEGs, only 4 DEGs coexpressed between the Ala group and Deh group; 48 DEGs coexpressed between Ala group and Mix group; and 634 DEGs coexpressed between Deh group and Mix group. A total of 247 significantly DEGs were shared by the Ala group, Deh group, and Mix group. (c) Heatmap showing the overlap of DEGs. The color means log2FC of the differential expression profiles. Blue represents genes with lower expression, and red represents genes with high expression.

A total of 15857 genes were detected in cultured microglia. 13,703 (NT), 13,709 (LPS), 13,809 (Ala), 13,997 (Deh), and 14,058 (Mix) genes were detected in the samples, and the standard of screening differential expressed genes (DEGs) was generally FDR ≤0.05. The numbers of upregulated and downregulated genes between samples are summarized in Figure 2(a). 1349 upregulated and 882 downregulated DEGs were identified from NT vs LPS in BV2 cells. Similarly, 1782, 2552, and 3132 upregulated and 2041, 2966, and 3443 downregulated in LPS vs Ala, LPS vs Deh, and LPS vs Mix, respectively. The expression level and statistics of the DEGs between samples are shown in Figures 2(b)–2(e).

**Figure 3:**
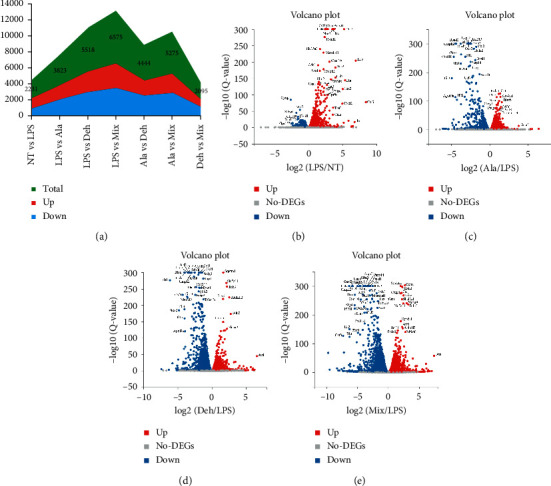
RNA-seq analysis of expression level and statistics of the differentially expressed genes (DEGs) between samples. DEGs were selected with FDR < 0.05. Up- and downregulated DEGs were colored with red and blue, respectively. (a) The number of upregulated and downregulated genes between samples. (b–e) Volcano plot of DEGs.

To obtain gene expression difference between samples obtained from cells treated with Ala, Deh, or Mix, the transcripts that attached a condition |log_2_FC| ≥1 were further analyzed. As a result (Figure 3(a)), the lowest number of DEGs was found in LPS vs Ala (Ala group), of which 157 upregulated and 221 downregulated. Meanwhile, 373 upregulated and 584 downregulated DEGs were found in LPS vs Deh (Deh group); and the largest number of DEGs was found in LPS vs Mix (Mix group), with 754 upregulated and 964 downregulated. In contrast, downregulated genes are higher than upregulated genes. There were 79, 72, and 789 expressed genes in the Ala group, Deh group, and Mix group, respectively (Figure 3(b)). Only 4 DEGs coexpressed between the Ala group and Deh group; 48 DEGs coexpressed between the Ala group and Mix group; and 634 DEGs coexpressed between the Deh group and Mix group. A total of 247 significantly DEGs were shared by the Ala group, Deh group, and Mix group. Results suggested that the number of DEGs between the Deh group and Mix group was larger than that between the Ala group and Mix group. The clustering heatmap exhibited the 247 DEGs (Figure 3(c)). In comparison, the number of upregulated genes by these compounds was lower than that of downregulated genes.

We selected some up- and downregulated genes after LPS treatment with or without Ala (or Deh or Mix) based on |log2FC| ≥1, FDR ≤0.05, and FPKM ≥100 and have listed them in Table 2. Stimulation of BV2 cells with LPS released cytokines such as interleukins (Il1*β*), colony stimulating factor (Csf3), chemokines (Ccl2, Ccl7, Cxcl10), tumor necrosis factor (Tnf*α*), and iNOS enzymes (Nos2), causing inflammation. After being treated with Ala or Deh or Mix, cells were downregulated the genes encoding cytokines and iNOS enzymes. For example, RNA-seq analysis revealed that the fold change (downregulated) of Il1*β* expression in Ala-, Deh-, and Mix-treated groups compared with the LPS group was −9.04, −69.67, and −867.61, respectively. Mix is the mixture of same concentration of Ala and Deh, but the effect of Mix group was more downregulated than the sum of them. Similarly, the fold change of Ccl7, Ccl2, Tnf*α*, and Nos2 expressions in the Mix group was more downregulated than that of the Ala and Deh group. All of Ala, Deh, and Mix upregulated 13 genes, such as Ddit3, Gclm, Trib3, and Srxn1. Among them, the fold change of upregulated genes in Mix was almost the sum of the fold change of upregulated genes in Ala and Deh. And either Ala or Deh upregulated genes are also listed in Table 2. The heatmap of these genes is presented in Figure S1.

### 3.4. GO Enrichment Analysis of DEGs

**Figure 4:**
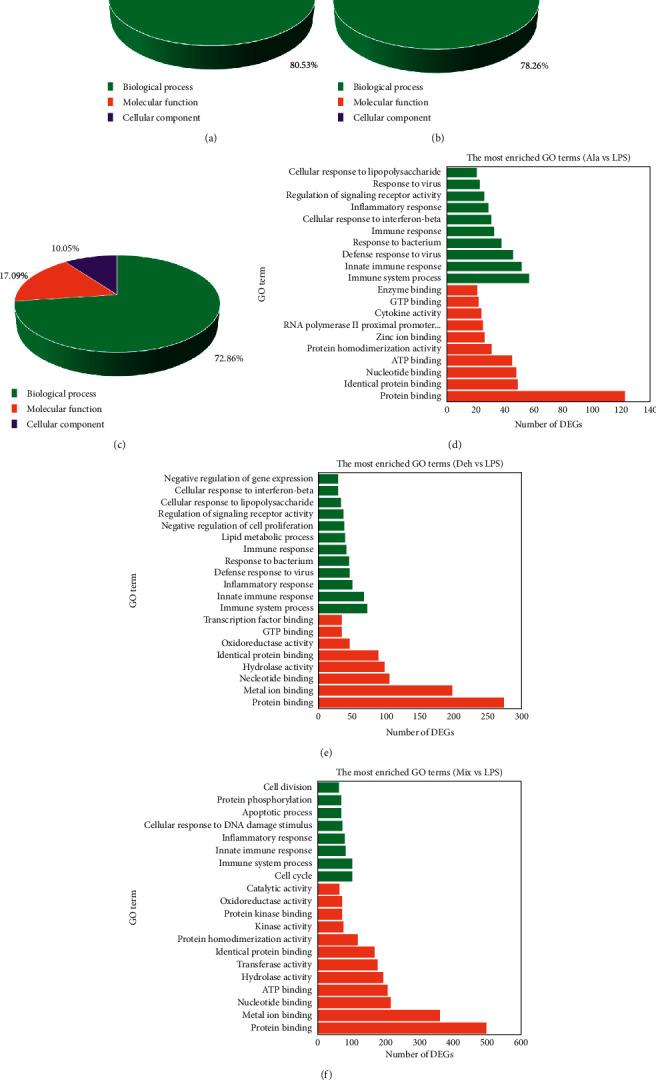
GO functional classification on DEGs. GO terms are divided into three ontologies: biological process, molecular function, and cellular component. (a–c) The GO functional classification of Ala group, Deh group, and Mix group. (d–f) The top 20 enriched GO terms of those groups. The *X*-axis indicated the number of genes in a category. All groups were involved in GO_BP including immune system process (GO:0002376), innate immune response (GO:0045087), and inflammatory response (GO:0006954) and GO_MF including protein binding (GO:0005515), nucleotide binding (GO:0000166), and identical protein binding (GO:0042802), respectively. (a) Ala vs LPS, (b) Deh vs LPS, and (c) Mix vs LPS.

A total of 190, 115, and 199 terms were significantly enriched in the Ala group, Deh group, and Mix group, respectively (FDR ≤0.05). The proportion of biological process (GO_BP), cellular component (GO_CC), and molecular function (GO_MF) in these groups are shown in Figures 4(a)–4(c). The top 20 enrichment of GO terms in the Ala group, Deh group, and Mix group were involved in similar biological process and molecular function, but some were different (Figures 4(d)–4(f)). In those top 20 GO terms, all groups were involved in GO_BP including immune system process (GO:0002376), innate immune response (GO:0045087), and inflammatory response (GO:0006954) and GO_MF including protein binding (GO:0005515), nucleotide binding (GO:0000166), and identical protein binding (GO:0042802), respectively. GO_MF included zinc ion binding (GO:0008270), RNA polymerase II proximal promoter sequence-specific DNA binding (GO:0000978) (binding), cytokine activity (GO:0005125) (binding), and enzyme binding (GO:0019899) (binding) and GO_BP included response to virus (GO:0009615) only in the Ala group. In addition, GO_MF included transcription factor binding (GO:0008134) and GO_BP included lipid metabolic process (GO:0006629), negative regulation of cell proliferation (GO:0008285), and negative regulation of gene expression (GO:0010629) only in the Deh group. Similarly, GO_MF included transferase activity (GO:0016740), kinase activity (GO:0016301), protein kinase binding (GO:0019901), and catalytic activity (GO:0003824) and GO_BP included cell cycle (GO:0007049), cellular response to DNA damage stimulus (GO:0006974), protein phosphorylation (GO:0006468), apoptotic process (GO:0006915), and cell division (GO:0051301) only in the Mix group.

### 3.5. KEGG Pathway Enrichment Analysis of DEGs

**Figure 5:**
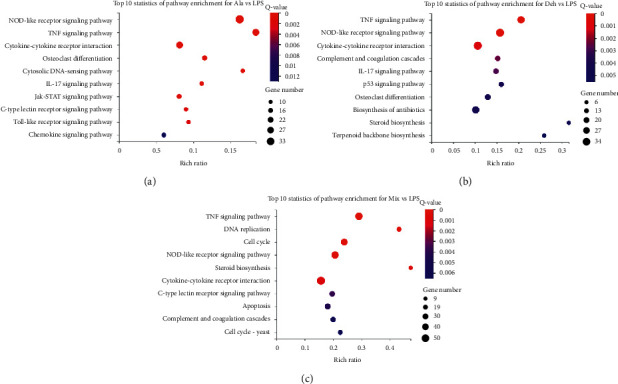
Scatter plots for KEGG enrichment results. (a) Top 10 statistic of enrichment pathway for LPS vs Ala (Ala group). (b) Top 10 statistic of enrichment pathway for LPS vs Deh (Deh group). (c) Top 10 statistic of enrichment pathway for LPS vs Mix (Mix group). *X*-axis Rich ratio means term candidate gene number/term gene number. All groups were significantly enriched in NOD-like receptor signaling pathway (pathway ID:04621), TNF signaling pathway (pathway ID:04668), and cytokine-cytokine receptor interaction (pathway ID:04060).

A total of 14, 20, and 30 pathways were significantly enriched in the Ala group, Deh group, and Mix group, respectively ((FDR ≤0.05) (Table S1). These pathways were primarily involved in immune system, cell growth and death, signal transduction, and signaling molecules and interaction. Top 10 statistic of enrichment pathway for the Ala group, Deh group, and Mix group are shown in Figure 5. The pathways significantly enriched in the Ala group mainly included NOD-like receptor signaling pathway (pathway ID:04621), TNF signaling pathway (pathway ID:04668), cytokine-cytokine receptor interaction (pathway ID:04060), osteoclast differentiation (pathway ID:04380), and cytosolic DNA-sensing pathway (pathway ID:04623). The pathways significantly enriched in the Deh group mainly included TNF signaling pathway (pathway ID:04668), NOD-like receptor signaling pathway (pathway ID:04621), cytokine-cytokine receptor interaction (pathway ID:04060), complement and coagulation cascades (pathway ID:04610), and IL-17 signaling pathway (pathway ID:04657). The pathways significantly enriched in the Mix group mainly included TNF signaling pathway (pathway ID:04668), DNA replication (pathway ID:03030), cell cycle (pathway ID:04110), NOD-like receptor signaling pathway (pathway ID:04621), and steroid biosynthesis (pathway ID:00100).

### 3.6. Validation of DEGs Data by Real-Time PCR

**Figure 6:**
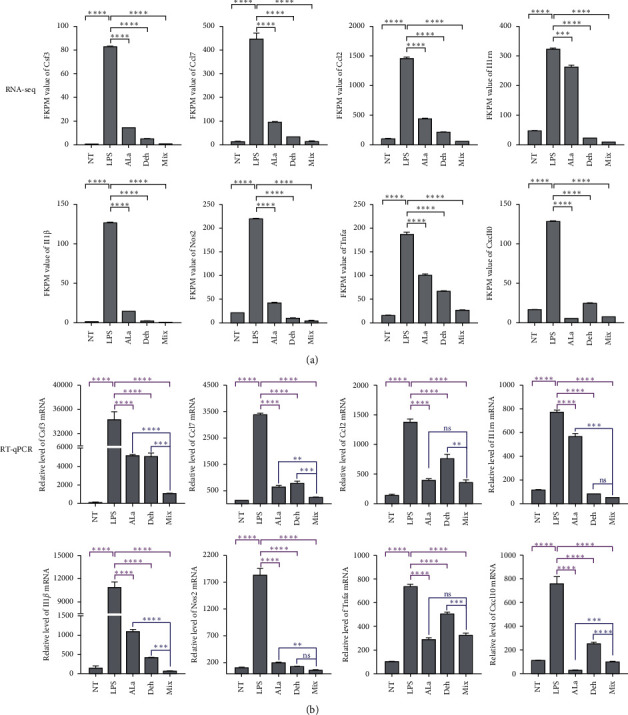
Validation of RNA-seq results using RT-qPCR. BV2 cells were pretreated with different concentrations of Ala, Deh, and Mix for 12 h then treated with LPS (100 ng/mL) for 6 h. (a) The FKPM value of *Csf*3, *Ccl*7, *Ccl*2, *Il*1*rn*, *Il1β*, *Nos*2, *Tnfα*, and *Cxcl*10 in RNA-seq. (b) The expression level of *Csf*3, *Ccl*7, *Ccl*2, *Il*1*rn*, *Il*1*β*, *Nos*2*, Tnfα*, and *Cxcl*10 in RT-qPCR. mRNA levels are expressed as percent of nontreated control (NT). Each value represents the mean ± standard deviation of duplicate cultures. ^∗∗∗∗^P <0.0001, ^∗∗∗^P <0.001, ^∗∗^P <0.01, and ns >0.05 indicate statistical significance.

To validate DEGs data, we treated BV2 cells with LPS with or without Ala, Deh, and Mix and analyzed the expression level of genes *Csf*3, *Ccl*7, *Ccl*2, *Il1rn*, *Il1β*, *Nos*2 (*iNOS*), *Tnfα*, and *Cxcl*10 by RT-qPCR. Stimulation of BV2 cells with LPS enhanced the expression of *Csf*3, *Ccl*7, *Ccl*2, *Il*1*rn*, *Il*1*β*, *Nos*2 (*iNOS*), *Tnfα*, and *Cxcl*10. The Ala, Deh, and Mix showed significant downregulation of all of these DEGs expression (Figures 6(a) and 6(b)). Nearly all of these downregulated DEGs shared the same results in Ala, Deh, and Mix groups.

**Figure 7:**
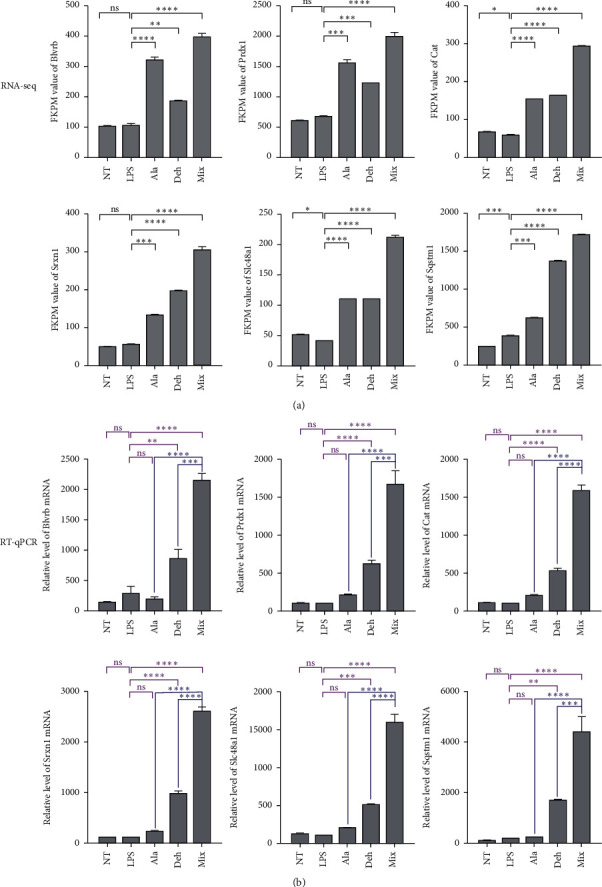
Validation of RNA-seq results using RT-qPCR. BV2 cells were pretreated with different concentrations of Ala, Deh, and Mix for 12 h and then treated with LPS (100 ng/mL) for 6 h. (a) The FKPM value of *Blvrb*, *Prdx*1, *Cat, Srxn*1, *Slc*48*a*1, and *Sqstm*1 in RNA-seq. (b) The expression level of *Blvrb*, *Prdx*1, *Cat*, *Srxn*1, *Slc*48*a*1, and *Sqstm*1 in RT-qPCR. mRNA levels are expressed as percent of nontreated control (NT). Each value represents the mean ± standard deviation of duplicate cultures. ^∗∗∗∗^P <0.0001, ^∗∗∗^P <0.001, ^∗∗^P <0.01, and ns >0.05 indicate statistical significance.

At the same time, we also analyzed the upregulated expression of genes *Blvrb*, *Prdx*1, *Cat*, *Srxn*1, *Slc*48*a*1, and *Sqstm*1 by RT-qPCR. Stimulation of BV2 cells with LPS had no significant difference in the expression of *Blvrb*, *Prdx*1, *Cat, Srxn*1, *Slc*48*a*1, and *Sqstm*1 genes. The Mix showed significant upregulation of *Blvrb*, *Prdx*1, *Cat*, *Srxn*1, *Slc*48*a*1, and *Sqstm*1 expression, compared with both Ala and Deh (Figure 7).

## 4. Discussion

In this study, we applied RNA-seq to systematically analyze the transcriptome of BV2 cells treated with Ala, Deh, and the Mix (mixture of Ala and Deh) after LPS stimulation. The RNA-seq gene expression profiles showed that the Deh group (957 DEGs) had significantly more DEGs than the Ala group (378 DEGs); however, the Mix group (1718 DEGs) had more DEGs than the Deh group (Figure 3). According to |log2FC| ≥1, FDR ≤0.05, and FPKM ≥100, top 9 downregulated and top 13 upregulated DEGs in both Ala and Deh were screened out. Among the downregulated DEGs, chemokines (*Ccl*7, *Ccl*2, and *Cxcl*10), interleukin (*Il1β*), tumor necrosis factor (*Tnfα*), colony stimulating factor (*Csf*3), and *Nos*2 enzyme were all proinflammatory factors. And most of the upregulated DEGs had antioxidant effects, such as *Sqstm*1, *Srxn*1, *Prdx*1, and *Cat*.

C-C motif chemokine ligand 2 (*Ccl*2) was upregulated in MCAO/R model rat brain tissue; downregulation of Ccl2 inhibited the inflammatory response and decreased the ischemic infarct area and also inhibited *Ccl*7, *Ccr*2, *Cxcl*16, and *Tnfα* expression [25]. Thymoquinone was one of the major compounds found in *Nigella sativa* and had anti-inflammatory effects that can attenuate the *Ccl*2, *Csf*3, *Cxcl*10, *Il*6, and other cytokines/chemokines released by BV2 microglia cells after stimulation of LPS [26]. C-C motif chemokine ligand 7 (*Ccl*7), also called monocyte chemotactic protein-3 (MCP-3), had been demonstrated to influence neutrophil chemotaxis [27]. Upregulation of *Ccl*7 recruits monocytes to the injury site, mediating local inflammatory responses that may associate with disease pathogenesis and clinical symptoms [28]. Colony stimulating factor 3 (granulocyte) (*Csf*3 or G-CSF) was upregulated by LPS stimulation in BV2 cells. In response to bacterial infections and cell-mediated immune responses, the production of *Csf*3 increases dramatically. *Csf*3, as a proinflammatory cytokine, can pass through the blood-brain barrier (BBB) and plays a neuroprotective role in stroke [29]. *Csf*3 had no significant regulatory role in the unactivated resting microglia, but LPS-stimulated microglia pretreated with *Csf*3 could significantly inhibit the NO expression level [30]. Subcutaneous injection of recombinant human *Csf*3 in spinal cord-hemisectioned mice can recruit microglia to the injured site, and *Csf*3 can inhibit the expression of proinflammatory factors and promote the expression of neurotrophic factors [31].

Sequestosome 1 (*Sqstm*1), also known as p62, is expressed in all of the tissues and regulates antioxidant response induced by the Keap1-Nrf2 system, autophagy, apoptosis, and inflammation [32]. In macrophages, CpdA, as a selective NR3C1/glucocorticoid receptor modulator, exhibited the anti-inflammatory effect through the mediator Sqstm1. The inhibition of LPS-induced *Il*6 and *Ccl*2 genes by CpdA was blocked by *Sqstm*1 silence [33]. Sulfiredoxin 1 (*Srxn*1), as a member of Sulfiredoxin antioxidant family, has antiapoptotic and neuroprotective effects to participate in oxidative stress. *Srxn*1 may be a potential target in the treatment of cerebral ischemia [34]. Knockdown of Srxn1 can promote LDH and MDA release and aggravate apoptosis of astrocytes, and upregulation of *Srxn1* inhibits the Notch signaling pathway and alleviates astrocytes H_2_O_2_-induced injury [35]. *Srxn*1 involved in Prdxs activity and protect PC12 cells from H_2_O_2_-induced oxidative stress [36]. Peroxiredoxins (Prxs or Prdxs) are a ubiquitous family of antioxidant enzymes, catalyzing the reduction of H_2_O_2_ and lipid hydroperoxides intracellular [36]. These enzymes have three major subtypes: typical 2-Cys Prxs (*Prdx*1–*Prdx*4), atypical 2-Cys Prxs (*Prdx*5), and 1-Cys Prxs (*Prdx*6) [37]. Nevertheless, extracellular Prxs trigger postischemic inflammation. Peroxidase (Prx) family proteins released from necrotic brain cells to cell extracellular induce the expression of inflammatory cytokines including interleukin 23 (Il23) in macrophages by activating Toll-like receptor 2 (TLR2) and TLR4, which promotes the death of nerve cells [38]. Catalase (*Cat*) was an enzyme that had two enzymatic activities (catalatic reaction and peroxidatic reaction) according to the concentration of H_2_O_2_ [39].

RNA sequencing found that Ala, Deh, and Mix mainly downregulate the proinflammatory genes and upregulate antioxidant genes, respectively. The results showed that these compounds may contribute to the neuroprotective effects through anti-inflammatory and antioxidant activities. The effect of group Mix was more significant than that of the other two groups Ala and Deh, and its effect was not simply the sum of the effects of Ala and Deh (Table 2, Figures 6 and 7). The Mongolian medicine Eerdun Wurile is a mixture of Ala, Deh, and other small molecules, so its effect is also not a simple single effect addition.

GO terms were grouped in three ontologies: biological process, molecular function, and cellular component. In the top 20 GO terms, compared with Ala and Deh, DEGs in Mix were mainly enriched in molecular function ontology, but DEGs in each group were mainly enriched in protein binding. In the biological process, the DEGs were mainly enriched in the term of immune system process in the Ala group and Deh group, while a large number of DEGs were mainly enriched in cell cycle in the Mix group (Figures 4(d)–4(f)). This indicated that the Mix had different effects from Ala and Deh in the biological process. The KEGG pathway analysis indicated that the DEGs were most enriched in NOD-like receptor signaling pathway, TNF signaling pathway, and cytokine-cytokine receptor interaction. DEGs in all three pathways were mainly chemokines and cytokines, such as *Ccl*2, *Ccl*12, *Cxcl*2, *Il*6, and *Il*1*β*. Although each group had similar enrichment pathways, DEGs were very different, indicating that Ala, Deh, and Mix trigger different effects. Compared with the Ala group and Deh group, the DEGs in the Mix group were differently enriched in DNA replication (pathway ID:03030), cell cycle (pathway ID:04110), apoptosis (pathway ID:04210), and cell cycle—yeast (pathway ID:04111) (Figure 5). DEGs in both DNA replication pathway and cell cycle pathway were Mxm2-7 and Pcna. Proliferating cell nuclear antigen (Pcna) was a cell cycle marker protein, which is well known to be an important part of eukaryotic chromosomal DNA replication and repair [40]. The minichromosome maintenance (Mcm) complex is in a ring shape, containing individual Mcm polypeptides (*Mcm*2-7). It was essential to initiating DNA replication and replication fork progression. During TBI, *Mcm*3 expression will be upregulated and promotes neuronal apoptosis [41]. The RNA-seq results showed that Mix significantly downregulated the expression of Mcm3 in LPS-stimulated BV2 cells.

To validate DEGs data, 8 inflammation-related cytokines and chemokines and 6 oxidation-related genes were selected for quantitative real-time PCR analysis. Nearly all of these down- and upregulated DEGs shared the same results in Ala, Deh, and Mix groups. RNA-seq and RT-qPCR results demonstrated that the Mix had the strongest downregulation effect on the proinflammatory cytokines including *Csf*3, *Ccl*7, *Ccl*2, *Il1rn*, *Il*1*β*, *Cxcl*10, *Tnfα*, and *Nos*2. At the same time, the Mix had remarkably increased the antioxidant genes including *Blvrb*, *Prdx*1, *Cat*, *Srxn*1, *Slc*48*a*1, and *Sqstm*1.

Traditional medicines such as Chinese and Mongolian medicines comprised of multiple components. The therapeutic mechanism of such medicine compound recipe relayed on the multiple biologically active molecules, which often simultaneously modulate multiple targets through multiple cellular pathways. In this experiment, RNA-seq was used to analyze the synergistic regulation effect of Ala and Deh (Mix) in BV2 microglial cells. Ala and Deh were found in *Inula helenium* L. and *Myristica fragrans* Houtt., the two main components of EW. We found that the synergistic effect of bioactive molecules is not simply the overlaying but also generate novel regulation effects that differ from both single molecules. Our data provide important evidence for further research on the therapeutic mechanism of traditional medicine including EW.

## Figures and Tables

**Table 1:** Summary of read numbers based on the RNA-seq data from NT, LPS, Ala, Deh, and Mix.

	Map to genome sequence									
	NT		LPS		Ala		Deh		Mix	
	NT_1	NT_2	LPS_1	LPS_2	Ala_1	Ala_2	Deh_1	Deh_2	Mix_1	Mix_2
Total reads (M)	47.33	47.33	47.33	47.33	47.33	47.33	47.33	47.33	47.33	45.57
Total clean reads (M)	44.83	44.95	44.81	45.43	44.84	44.59	44.7	45.24	45.06	44.47
Mapped reads (%)	89.97	89.49	89.67	90.51	91.18	91.12	90.21	90.92	91.07	92.15
Unique match (%)	80.44	80.23	80.46	81.01	81.96	81.93	81.33	82.04	82.34	83.35

**Table 2:** Some upregulated and downregulated genes list.

Gene ID	Gene symbol	Description	FC (LPS/NT)	FC (Ala/LPS)	FC (Deh/LPS)	FC (Mix/LPS)
Both Ala and Deh downregulated genes						
16176	*Il1β*	*Interleukin 1 beta*	125.37	−9.04	−69.67	−867.61
12985	*Csf3*	*Colony stimulating factor 3 (granulocyte)*	349.71	−5.91	−19.37	−162.31
20306	*Ccl7*	*Chemokine (C-C motif) ligand 7*	38.05	−4.80	−15.56	−81.64
20296	*Ccl2*	*Chemokine (C-C motif) ligand 2*	15.89	−3.37	−7.81	−32.08
18126	*Nos2*	*Nitric oxide synthase 2, inducible*	11.63	−5.34	−28.10	−73.27
23962	*Oasl2*	*2′-5′ Oligoadenylate synthetase-like 2*	2.60	−13.91	−10.18	−54.89
16181	*Il1rn*	*Interleukin 1 receptor antagonist*	7.31	−1.24	−16.36	−42.59
21926	*Tnf*	*Tumor necrosis factor*	13.09	−1.87	−3.15	−8.05
15945	*Cxcl10*	*Chemokine (C-X-C motif) ligand 10*	8.63	−25.76	−6.03	−21.95

Both Ala and Deh upregulated genes						
13198	*Ddit3*	*DNA-damage inducible transcript 3*	1.58	1.72	6.77	9.58
14630	*Gclm*	*Glutamate-cysteine ligase, modifier subunit*	1.23	3.33	2.52	6.45
228775	*Trib3*	*Tribbles pseudokinase 3*	1.31	4.65	2.10	5.56
76650	*Srxn1*	*Sulfiredoxin 1 homolog (S. cerevisiae)*	1.21	3.13	2.36	4.85
107503	*Atf5*	*Activating transcription factor 5*	1.13	3.86	2.12	4.84
67739	*Slc48a1*	*Solute carrier family 48 (heme transporter), member 1*	−1.17	2.40	2.67	4.62
12359	*Cat*	*Catalase*	−1.08	2.48	2.63	4.51
72017	*Cyb5r1*	*Cytochrome b5 reductase 1*	1.19	1.53	3.14	4.08
433375	*Creg1*	*Cellular repressor of E1A-stimulated genes 1*	−1.17	2.19	2.17	4.05
18412	*Sqstm1*	*Sequestosome 1*	1.68	1.60	3.16	4.00
233016	*Blvrb*	*Biliverdin reductase B (flavin reductase (NADPH))*	1.10	3.02	1.56	3.36
16889	*Lipa*	*Lysosomal acid lipase A*	−1.09	2.07	1.56	2.93
18477	*Prdx1*	*Peroxiredoxin 1*	1.19	2.31	1.62	2.68

Either Ala or Deh upregulated genes						
12282	*Hyou1*	*Hypoxia upregulated 1*	1.01	−1.16	1.48	2.23
12491	*Cd36*	*CD36 molecule*	−1.03	2.79	−1.29	3.17
67951	*Tubb6*	*Tubulin, beta 6 class V*	1.21	1.09	−2.63	−3.41
13132	*Dab2*	*Disabled 2, mitogen-responsive phosphoprotein*	1.28	1.11	−2.92	−3.71
19241	*Tmsb4x*	*Thymosin, beta 4, X chromosome*	1.21	1.14	−1.96	−2.51
20250	*Scd2*	*Stearoyl-Coenzyme A desaturase 2*	−1.30	1.16	−2.82	−3.34
18793	*Plaur*	*Plasminogen activator, urokinase receptor*	2.64	1.21	−2.83	−3.65
11655	*Alas1*	*Aminolevulinic acid synthase 1*	2.11	1.21	−2.20	−2.43
71602	*Myo1e*	*Myosin IE*	1.73	1.24	−2.50	−3.14
11465	*Actg1*	*Actin, gamma, cytoplasmic 1*	1.35	1.25	−2.75	−3.72
20750	*Spp1*	*Secreted phosphoprotein 1*	1.22	1.25	−2.41	−1.75
12306	*Anxa2*	*Annexin A2*	1.04	1.25	−2.28	−2.11
11770	*Fabp4*	*Fatty acid binding protein 4, adipocyte*	1.77	1.28	−3.58	−5.02
326618	*Tpm4*	*Tropomyosin 4*	1.53	1.28	−1.68	−2.14
16956	*Lpl*	*Lipoprotein lipase*	−1.08	1.32	−2.74	−1.82
20620	*Plk2*	*Polo like kinase 2*	1.87	1.38	−2.09	−1.97
16985	*Lsp1*	*Lymphocyte specific 1*	−1.27	1.39	−2.33	−3.53
17381	*Mmp12*	*Matrix metallopeptidase 12*	2.06	1.51	−11.69	−4.98
16952	*Anxa1*	*Annexin A1*	1.22	1.59	−1.85	−2.18
29818	*Hspb7*	*Heat shock protein family, member 7 (cardiovascular)*	−1.04	1.62	−4.47	−4.06
18792	*Plau*	*Plasminogen activator, urokinase*	1.17	1.90	−3.70	−5.66

## Data Availability

The data used to support the findings of this study are included within the Supplementary Materials file.

## Supplementary Materials

Supplementary Materials The following are available online: *Table S1*. Kyoto Encyclopedia of Genes and Genomes (KEGG) enriched by DEGs among all three compared groups (LPS vs Ala, LPS vs Deh, LPS vs Mix). *Figure S1*. Analysis of gene expression based on |log2FC| ≥1, FDR ≤0.05, and FPKM ≥100. (a) Heatmap of both Ala and Deh downregulated DEGs. (b) Heatmap of both Ala and Deh upregulated DEGs. (c) Heatmap of either Ala or Deh upregulated DEGs.
