# Supplementary material for: Synergistic Regulation of Microglia Gene Expression by Natural Molecules in Herbal Medicine
Source: Evid Based Complement Alternat Med. 2021 Aug 18;2021:9920364. doi: 10.1155/2021/9920364 (PMC8390137; doi:10.1155/2021/9920364)
Supplement: Supplementary Materials — The following are available online: Table S1. Kyoto Encyclopedia of Genes and Genomes (KEGG) enriched by DEGs among all three compared groups (LPS vs Ala, LPS vs Deh, LPS vs Mix). Figure S1. Analysis of gene expression based on |log2FC| ≥1, FDR ≤0.05, and FPKM ≥100. (a) Heatmap of both Ala and Deh downregulated DEGs. (b) Heatmap of both Ala and Deh upregulated DEGs. (c) Heatmap of either Ala or Deh upregulated DEGs. [file 9920364.f1.docx]

Article

Synergistic regulation of microglia gene expression by natural molecules in herbal medicine

Qiburi Qiburi^1^, Temuqile Temuqile^2,^*, Huricha Baigude^1,2,^*

^1^ Institute of Mongolian Medicinal Chemistry, School of Chemistry & Chemical Engineering, Inner Mongolia University, Hohhot, Inner Mongolia, 010020, China; [qiburi@mail.imu.edu.cn(Q.Q.)](mailto:qiburi@mail.imu.edu.cn(Q.Q.));

^2^ International Hospital of Mongolian Medicine, Hohhot, Inner Mongolia, 010021, China;

* Correspondence: [Tmqyx01@gmail.com](mailto:Tmqyx01@gmail.com) (T.T.); [hbaigude@imu.edu.cn](mailto:hbaigude@imu.edu.cn) (H.B.); Tel.: +86-471-5182005 (T.T.); Tel.: +86-471-4993165 (H.B.)

**Supplementary Table S1.** Kyoto Encyclopedia of Genes and Genomes (KEGG) enriched by DEGs among all three compared group (Ala vs LPS, Deh vs LPS, Mix vs LPS)

| **Pathway ID** | **Pathway** | **DEGs Num** | ***Q* value (FDR)** |
| --- | --- | --- | --- |
| **Ala group** |  |  |  |
| 04621 | NOD-like receptor signaling pathway | 33 | 2.79E-18 |
| 04668 | TNF signaling pathway | 26 | 9.02E-16 |
| 04060 | Cytokine-cytokine receptor interaction | 26 | 1.66E-07 |
| 04380 | Osteoclast differentiation | 16 | 1.52E-06 |
| 04623 | Cytosolic DNA-sensing pathway | 11 | 4.09E-06 |
| 04657 | IL-17 signaling pathway | 12 | 8.26E-05 |
| 04630 | Jak-STAT signaling pathway | 14 | 4.44E-04 |
| 04625 | C-type lectin receptor signaling pathway | 11 | 0.001193901 |
| 04620 | Toll-like receptor signaling pathway | 10 | 0.001727801 |
| 04062 | Chemokine signaling pathway | 13 | 0.01207378 |
| 04640 | Hematopoietic cell lineage | 9 | 0.01207378 |
| 04622 | RIG-I-like receptor signaling pathway | 7 | 0.01427817 |
| 04917 | Prolactin signaling pathway | 7 | 0.01802629 |
| 04512 | ECM-receptor interaction | 7 | 0.04334925 |
| **Deh group** |  |  |  |
| 04668 | TNF signaling pathway | 29 | 4.87E-09 |
| 04621 | NOD-like receptor signaling pathway | 32 | 2.95E-07 |
| 04060 | Cytokine-cytokine receptor interaction | 34 | 8.84E-04 |
| 04610 | Complement and coagulation cascades | 16 | 0.002395464 |
| 04657 | IL-17 signaling pathway | 16 | 0.002725816 |
| 04115 | p53 signaling pathway | 13 | 0.004181192 |
| 04380 | Osteoclast differentiation | 18 | 0.004181192 |
| 01130 | Biosynthesis of antibiotics | 28 | 0.004436363 |
| 00100 | Steroid biosynthesis | 6 | 0.005171001 |
| 00900 | Terpenoid backbone biosynthesis | 7 | 0.005358887 |
| 01110 | Biosynthesis of secondary metabolites | 40 | 0.00828782 |
| 04064 | NF-kappa B signaling pathway | 15 | 0.0141156 |
| 04625 | C-type lectin receptor signaling pathway | 15 | 0.0141156 |
| 04145 | Phagosome | 22 | 0.0182876 |
| 04210 | Apoptosis | 17 | 0.0182876 |
| 04623 | Cytosolic DNA-sensing pathway | 10 | 0.0182876 |
| 04640 | Hematopoietic cell lineage | 14 | 0.0182876 |
| 04062 | Chemokine signaling pathway | 21 | 0.02337917 |
| 04620 | Toll-like receptor signaling pathway | 13 | 0.02372584 |
| 04979 | Cholesterol metabolism | 8 | 0.03855552 |
| **Mix group** |  |  |  |
| 04668 | TNF signaling pathway | 41 | 3.20E-10 |
| 03030 | DNA replication | 16 | 2.41E-06 |
| 04110 | Cell cycle | 33 | 3.87E-06 |
| 04621 | NOD-like receptor signaling pathway | 42 | 3.95E-06 |
| 00100 | Steroid biosynthesis | 9 | 5.49E-04 |
| 04060 | Cytokine-cytokine receptor interaction | 50 | 9.24E-04 |
| 04625 | C-type lectin receptor signaling pathway | 24 | 0.003621542 |
| 04210 | Apoptosis | 28 | 0.004139188 |
| 04610 | Complement and coagulation cascades | 21 | 0.005937001 |
| 04111 | Cell cycle - yeast | 16 | 0.006185602 |
| 04620 | Toll-like receptor signaling pathway | 21 | 0.006185602 |
| 04640 | Hematopoietic cell lineage | 22 | 0.006185602 |
| 04630 | Jak-STAT signaling pathway | 29 | 0.007374356 |
| 04115 | p53 signaling pathway | 17 | 0.00835017 |
| 04064 | NF-kappa B signaling pathway | 22 | 0.01160933 |
| 04657 | IL-17 signaling pathway | 20 | 0.01330458 |
| 00240 | Pyrimidine metabolism | 20 | 0.01412501 |
| 01130 | Biosynthesis of antibiotics | 39 | 0.0176913 |
| 04623 | Cytosolic DNA-sensing pathway | 14 | 0.0176913 |
| 00900 | Terpenoid backbone biosynthesis | 8 | 0.01841207 |
| 04145 | Phagosome | 33 | 0.01841207 |
| 03410 | Base excision repair | 10 | 0.0241431 |
| 01110 | Biosynthesis of secondary metabolites | 59 | 0.02576181 |
| 04068 | FoxO signaling pathway | 23 | 0.02576181 |
| 03430 | Mismatch repair | 7 | 0.03365316 |
| 04380 | Osteoclast differentiation | 22 | 0.03778612 |
| 00480 | Glutathione metabolism | 15 | 0.03835479 |
| 04979 | Cholesterol metabolism | 11 | 0.03848984 |
| 04113 | Meiosis - yeast | 12 | 0.04085015 |
| 00983 | Drug metabolism - other enzymes | 17 | 0.04754294 |


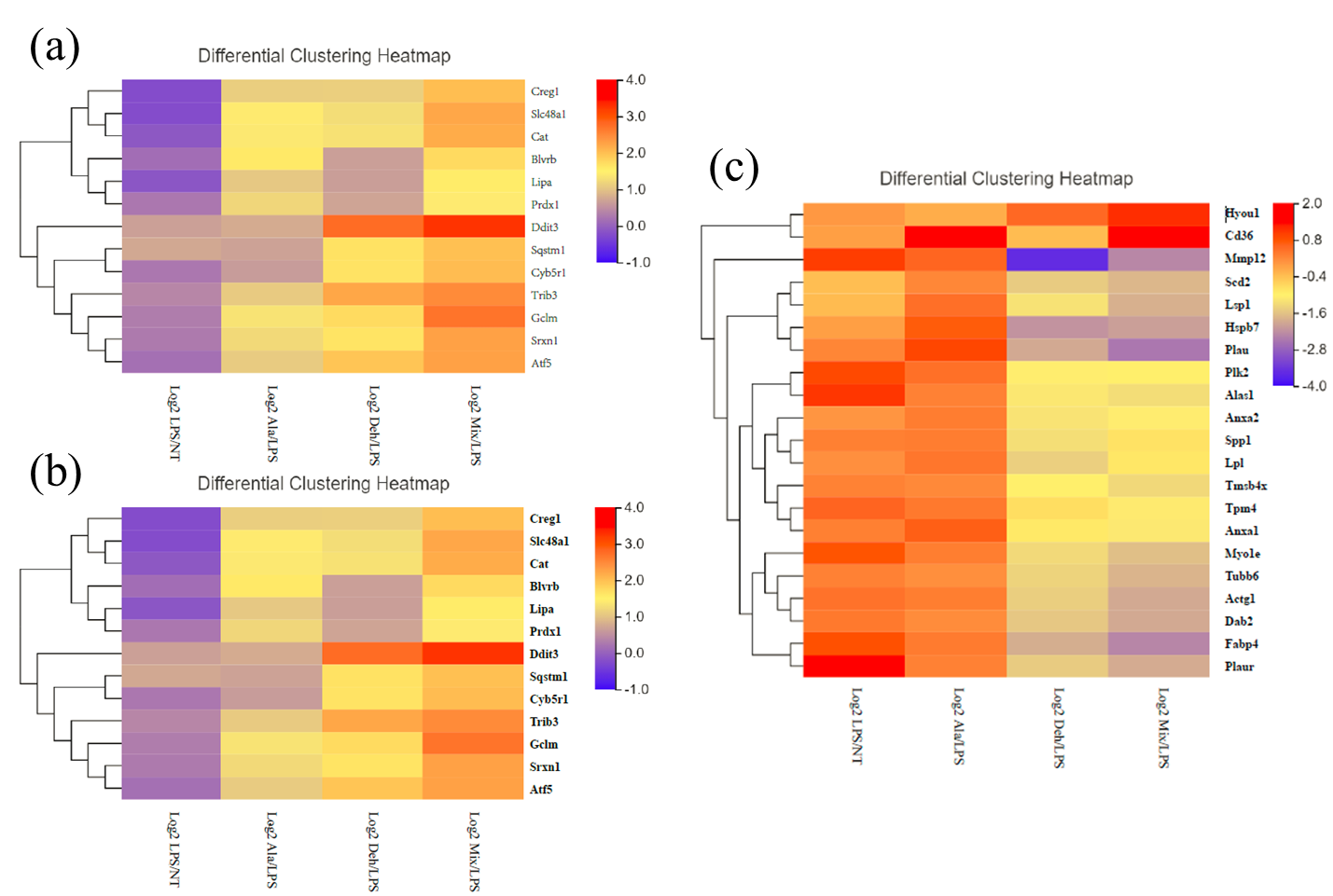


**Supplementary Figure 1.** Analysis of gene expression based on |log2FC|≥1, FDR≤0.05 and FPKM≥100. (A) Heatmap of both Ala and Deh down-regulated DEGs. (B) Heatmap of both Ala and Deh up-regulated DEGs. (C) Heatmap of either Ala or Deh up-regulated DEGs.
